# The Impact of Antarctic Ice Microalgae Polysaccharides on D-Galactose-Induced Oxidative Damage in Mice

**DOI:** 10.3389/fnut.2021.651088

**Published:** 2021-03-09

**Authors:** Ruokun Yi, Lei Deng, Jianfei Mu, Chong Li, Fang Tan, Xin Zhao

**Affiliations:** ^1^Chongqing Collaborative Innovation Center for Functional Food, Chongqing University of Education, Chongqing, China; ^2^Chongqing Engineering Research Center of Functional Food, Chongqing University of Education, Chongqing, China; ^3^Chongqing Engineering Laboratory for Research and Development of Functional Food, Chongqing University of Education, Chongqing, China; ^4^Department of Gastroenterology and Hepatology, Chongqing University Central Hospital (Chongqing Emergency Medical Center), Chongqing, China; ^5^Department of Public Health, Our Lady of Fatima University, Valenzuela, Philippines

**Keywords:** Antarctic ice microalgae, polysaccharide, D-galactose, oxidative damage, mice

## Abstract

Antarctic ice microalgae (*Chlamydomonas* sp.) are a polysaccharide-rich natural marine resource. In this study, we evaluated the impact of Antarctic ice microalgae polysaccharides (AIMP) on D-galactose-induced oxidation in mice. We conducted biological and biochemical tests on tissue and serum samples from mice treated with AIMP. We found that AIMP administration was associated with improved thymus, brain, heart, liver, spleen, and kidney index values. We also found that AIMP treatment inhibited the reduced aspartate aminotransferase, alanine aminotransferase, alkaline phosphatase, superoxide dismutase, glutathione peroxidase, and glutathione levels as well as the increased serum, splenic, and hepatic nitric oxide and malondialdehyde levels arising from oxidation in these animals. Pathological examination revealed that AIMP also inhibited D-galactose-induced oxidative damage to the spleen, liver, and skin of these animals. AIMP was additionally found to promote the upregulation of neuronal nitric oxide synthase, endothelial nitric oxide synthase, cuprozinc-superoxide dismutase, manganese superoxide dismutase, catalase, heme oxygenase-1, nuclear factor erythroid 2-related factor 2, γ-glutamylcysteine synthetase, and NAD(P)H dehydrogenase [quinone] 1 as well as the downregulation of inducible nitric oxide synthase in these animals. High-performance liquid chromatography analysis revealed AIMP to be composed of five monosaccharides (mannitol, ribose, anhydrous glucose, xylose, and fucose). Together, these results suggest that AIMP can effectively inhibit oxidative damage more readily than vitamin C in mice with D-galactose-induced oxidative damage, which underscores the value of developing AIMP derivatives for food purposes.

## Introduction

Antarctic ice microalgae (*Chlamydomonas* sp.) are found in both fixed and floating ice in the Antarctic sea. Due to the extreme and prolonged cold in this region, Antarctic ice microalgae have significant physiological and morphological differences when compared to other forms of algae. Antarctic ice microalgae are collagen-rich, yellow-to-brown organisms that contain high levels of calcium, iron, iodine, phosphorus, and cellulose, and their mineral element content levels are higher than those in *Chlamydomonas monadina* (1). Studies of these microalgae began in the 1960s (2), and recent work has resulted in more deeply characterization of the biochemical and physiological properties of these organisms. In traditional Chinese medicine, Antarctic ice microalgae are considered to protect the skin and soften the blood vessels (3). However, up to now, few studies have evaluated the biological activity of Antarctic ice microalgae. At present, only a single study has shown that there are antioxidant enzymes in Antarctic ice algae, which may have antioxidant effects (4). However, other microalgae have been shown to have antioxidant effects. 2, 2 diphenyl-1-picrylhydrazyl (DPPH) test and 2,2′-azino-bis (ethylbenzthiazoline-6-sulfonic acid (ABTS.^+^) experiments showed that the extracts of some microalgae had good ability of scavenging free radicals *in vitro*, and they were antioxidants (5). In addition, *in vitro* study also confirmed that a variety of microalgae were non-toxic and had inhibitory effect on DNA oxidative damage and cell oxidative damage caused by H_2_O_2_ (6). As one of the most widely used microalgae, *Arthrospira platensis* has been confirmed by animal experiments and clinical experiments to have antioxidant effect and regulate a variety of diseases including hypercholesterolemia, hypertriglyceridemia, cardiovascular disease and inflammatory disease through its antioxidant capacity (7). At the same time, a variety of microalgae also have good antioxidant effect, such as *Tetraselmis suecica, Botryococcus braunii, Neochloris oleoabundans* and so on, they all contain polysaccharide substances and have the potential as a source of natural antioxidants (8). Meanwhile, polysaccharides as important bioactive substances have been confirmed to have antioxidant and anti-inflammatory effects through *in vitro* cell studies and *in vivo* animal experiments (9–11). The active ingredients in some seaweeds can be used to develop antioxidant foods and drugs. However, in terms of traditional health care products, Antarctic ice algae still lack scientific evidence proving their biological activity, which hinders their development and use. This study is the first to analyze the antioxidant effectiveness of Antarctic ice microalgae as medicinal and health care products.

Normal homeostasis depends on balanced oxidative and antioxidative activity *in vivo*. A small amount of reactive oxygen species (ROS) can clear viruses from the body, but a large amount of ROS in the body will cause oxidative damage (12, 13). The intake of nutrients can effectively reduce lipid peroxidation and oxidative stress (14).

D-galactose can establish an effective oxidative damage state in animals (15). In this study, we also used D-galactose to establish an oxidative damage model in mice to test the antioxidant effect of Antarctic ice algae polysaccharide (AIMP) and its regulatory effect on HO-1, the key expression of oxidation. The impacts of AIMP on serum and tissue changes in these animals were examined. By assessing AIMP-induced changes in the expression levels of oxidation-related genes, we evaluated the mechanisms whereby AIMP inhibits oxidative stress, thus providing a theoretical basis for future studies regarding the use and pharmaceutical development of AIMP.

## Materials and Methods

### AIMP Extraction and Purification

Antarctic ice algae are mainly found off the southern coast of Chile, and the samples used in this study were from this area. Initially, a sample (500 g) of Antarctic ice microalgae (Qingdao Shenshoutang Seafood Co., Ltd., Qingdao, Shandong, China) was ultrasonically extracted in 2.5 L of ddH_2_O for 40 min at 90°C to facilitate polysaccharide extraction. The resultant solution was filtered through sterile gauze. The filtered residue was collected, resuspended in water, and ultrasonically extracted (CYS-Y21S Ultrasonic Extractor, Hangzhou Supersonic Electromechanical Technology Co., Ltd., Hangzhou, China) at 600W for 20 min. These steps were repeated thrice. The three extract preparations were combined, freeze-dried, and resuspended in a small volume of hot water. Next, ethanol was added to a final concentration of 80% EtOH. The samples were then freeze-dried for 8 h. The samples were then again resuspended in water and 80% EtOH and allowed to stay overnight at 4°C. The samples were then freeze-dried a final time to isolate the crude polysaccharides. The Sevag method was then used four times to isolate the crude polysaccharides dissolved in hot water (16). These four aqueous samples were subsequently combined. A small quantity of activated carbon was added to this solution, which was then allowed to stay for 10 min prior to filtration. EtOH was added to the filtrate to a final concentration of 80% EtOH, and the samples were again allowed to stay at 4°C overnight. The final refined AIMP preparation was obtained by fully freeze-drying the samples and then transferring them to a vacuum drying oven for 4 h.

### Animal Model

**Figure 1:**
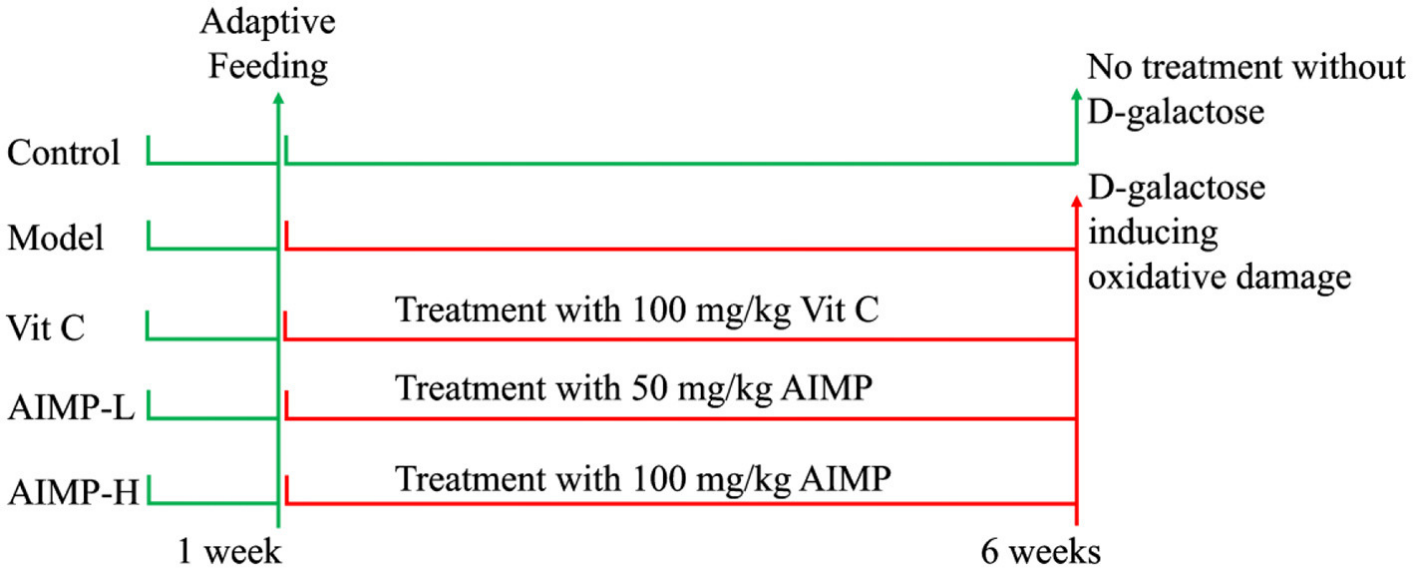
Implementation process of animal experiment in this study.

We obtained fifty 6-week-old ICR mice (25 males, 25 females; SPF-grade, Chongqing Medical University, Chongqing, China). The animals were allowed to acclimatize for 1 week prior to being assigned at random to one of five different treatment groups (*n* = 10/group; five males, five females/group): a control group, a model group (oxidative damage control group), a low-dose AIMP group (AIMP-L), a high-dose AIMP group (AIMP-H), and a vitamin C treatment group (Vit C). The mice in the control and model groups were fed standard food and water for 6 weeks. The mice in the AIMP-L and AIMP-H groups were treated with 50 mg/kg or 100 mg/kg of AIMP, respectively, by gavage each day for 6 weeks. During this same 6-week period, the mice in the Vit C group were treated with 100 mg/kg of Vit C by gavage each day. In these 6 weeks, the mice in each group except for the control group were also intraperitoneally injected once per day with 120 mg/kg of D-galactose (Figure 1). After the treatment period ended, the mice were fasted for 24 h before being euthanized (17). Blood samples were collected from the hearts of the mice, and liver and splenic tissue samples were collected for downstream analyses. Thymus, brain, heart, liver, spleen, and kidney organ index values were determined as follows:

$$\begin{array}{l} \text{organ index}=\text{organ weight }\left(\text{mg}\right)/\text{mouse body weight }\left(\text{g}\right). \end{array}$$

### Assessment of ALT, AST, AKP, NO, GSH, and MDA Levels and SOD and GSH-Px Activity

Blood samples from all mice were spun for 10 min at 4,000 rpm. The serum was then isolated and used to assess the levels of alanine aminotransferase (ALT), aspartate aminotransferase (AST), alkaline phosphatase (AKP), nitric oxide (NO), glutathione (GSH) and malondialdehyde (MDA) and the activity of superoxide dismutase (SOD) and glutathione peroxidase (GSH-Px) using appropriate test kits (Nanjing Jiancheng Bioengineering Institute, Nanjing, Jiangsu, China). The 10 μL serum of each mouse were determined these indexes used the kits and finally, the indexes were calculated by the absorbance. For the hematic samples, 10% homogenates were prepared prior to spinning for 10 min at 4,000 rpm. The supernatants were then collected, and the levels of NO, GSH and MDA and the activity of SOD and GSH-Px were assessed using appropriate test kits (Nanjing Jiancheng Bioengineering Institute, Nanjing, Jiangsu, China).

### Assessment of Hepatic and Splenic Morphology

Murine hepatic and splenic tissue samples (~0.5 cm^3^) were isolated, fixed for 48 h with 10% formalin, embedded in paraffin, sectioned, and stained with hematoxylin and eosin. A light microscope (BX43, Olympus, Tokyo, Japan) was then used to evaluate tissue morphology.

### Quantitative PCR

RNAzol (Invitrogen, Carlsbad, CA, USA) was used to extract the total RNA from the hepatic and splenic tissue homogenates. The RNA was diluted to a concentration of 1 μg/μL with enzyme-free water, and then 1 μL of the diluted RNA and 9 μL of oligo (dT) primer were put into the polymerase chain reaction (PCR) apparatus at 65°C for 5 min to complete a thermal cycle. Next, 4 μL of 5× reaction buffer, 1 μL of RiboLock RNase inhibitor, 2 μL of 100 mM dNTP mix, and 1 μL of RevertAid Reverse Transcriptase were added and then mixed. The cDNA template was generated via PCR at 42°C for 60 min and 70°C for 5 min. Next, 2 μL of this cDNA template per sample was combined with 10 μL of SYBR Green PCR Master Mix and 1 μL of forward and reverse primers (Thermo Fisher Scientific, Waltham, MA, USA; Table 1). The following thermocycler settings were used: 95°C for 60 s, 40 cycles at 95°C for 15 s, 55°C for 30 s, and 72°C for 33 s. GAPDH was used to normalize gene expression, with the 2^−ΔΔCt^ method being used to assess relative gene expression (StepOnePlus Real-Time PCR System, Thermo Fisher Scientific, Waltham, MA, USA) (18).

**Table 1:** Primer sequences used in this study.

**Gene name**	**Sequence**
nNOS	Forward: 5′-ACGGCAAACTGCACAAAGC-3′
	Reverse: 5′-CGTTCTCTGAATACGGGTTGTTG-3′
eNOS	Forward: 5′-TCAGCCATCACAGTGTTCCC-3′
	Reverse: 5′-ATAGCCCGCATAGCGTATCAG-3′
iNOS	Forward: 5′-GTTCTCAGCCCAACAATACAAGA-3′
	Reverse: 5′-GTGGACGGGTCGATGTCAC-3′
Cu/Zn-SOD	Forward: 5′-AACCAGTTGTGTTGTCAGGAC-3′
	Reverse: 5′-CCACCATGTTTCTTAGAGTGAGG-3′
Mn-SOD	Forward: 5′-CAGACCTGCCTTACGACTATGG-3′
	Reverse: 5′-CTCGGTGGCGTTGAGATTGTT-3′
CAT	Forward: 5′- GGAGGCGGGAACCCAATAG−3′
	Reverse: 5′- GTGTGCCATCTCGTCAGTGAA-3′
HO-1	Forward: 5′-ACAGATGGCGTCACTTCG-3′
	Reverse: 5′-TGAGGACCCACTGGAGGA-3′
Nrf2	Forward: 5′-CAGTGCTCCTATGCGTGAA-3′
	Reverse: 5′-GCGGCTTGAATGTTTGTC-3′
γ-GCS	Forward: 5′-GCACATCTACCACGCAGTCA-3′
	Reverse: 5′-CAGAGTCTCAAGAACATCGCC-3′
NQO1	Forward: 5′-CTTTAGGGTCGTCTTGGC-3′
	Reverse: 5′-CAATCAGGGCTCTTCTCG-3′
GAPDH	Forward: 5′-AGGTCGGTGTGAACGGATTTG-3′
	Reverse: 5′-GGGGTCGTTGATGGCAACA-3′

### Western Blotting

According to the experimental method of Liu et al. (19), 1 mL of radioimmunoprecipitation assay buffer containing 10 μL of phenylmethylsulfonyl fluoride (Thermo Fisher Scientific, MA, USA) was used to lyse the hepatic and splenic tissue sections for 5 min. They were then spun for 15 min at 12,000 rpm and 4°C. The protein levels in these samples were assessed via bicinchoninic acid assay (Thermo Fisher Scientific), after which they were diluted to 50 μg/mL. The samples were then mixed with sample buffer (4:1) and denatured for 5 min by heating to 100°C. They were then chilled on ice for 5 min. The samples were then separated via sodium dodecyl sulfate-polyacrylamide gel electrophoresis using previously prepared gels, with a pre-stained protein ladder used for reference. The proteins were next transferred to polyvinylidene fluoride membranes that were then blocked for 1 h using 5% non-fat milk in TBST. The blots were then incubated with 1 μg/mL primary antibodies of nNOS, eNOS, iNOS, Cu/Zn-SOD, Mn-SOD, CAT, HO-1, Nrf2 and NQO1 at 25°C for 2 h. They were then washed five times with TBST and then incubated with 0.4 μg/mL Goat anti-Mouse IgG secondary antibodies (Thermo Fisher Scientific) at 25°C for 1 h. SuperSignal West Pico PLUS reagent was then used to detect the protein bands with an imaging system (Tanon 5200, Shanghai Tanon Technology Co., Ltd., Shanghai, China).

### Derivative Product Preparation

Initially, standard mixtures of monosaccharides were prepared by mixing 100 μL of 0.5 mol/L PMP-methanol and 100 μL of 0.3 mol/L NaOH with 100 μL of the mixed standard solution of each monosaccharide. This solution was mixed for 1 min, then the derivatization reaction was conducted for 40 min at 60°C in a water bath. The samples were then cooled to room temperature, and 100 μL of 0.3 mol/L sample was used. Next, HCl was neutralized in this solution, and 2 mL of chloroform was added. The supernatants were collected and extracted thrice via vortexing for 1 min. An AIMP hydrolysate sample was also derived via this same approach.

### High-Performance Liquid Chromatography

The AIMP was initially dissolved to 10 mg/mL using dimethyl sulfoxide, then it was diluted to 2 mg/mL using 50% methanol. The AIMP samples and standard solutions were then passed through a 0.22-μm membrane, and 10 μL of each solution was subjected to high-performance liquid chromatography analysis. The mobile phase for this analysis was composed of an ammonium acetate buffer solution (A) (0.05 mol/L, pH=5.5) and acetonitrile (B). A 10-μL injection volume was used, and the column was maintained at 30°C with a flow rate of 1.0 mL/min and a wavelength of 250 nm. The gradient settings were: 0 to 30 min, 1-18% B; 30 to 50 min, 18-25% B; and 50 to 60 min, 25-30% B (UltiMate 3000, Thermo Fisher Scientific, Inc., Waltham, MA, USA).

### Statistical Analysis

All samples were analyzed in triplicate. SAS 9.1 software (SAS Institute Inc., Cary, NC, USA) was used for all statistical testing. Data were compared via one-way analysis of variance, with *p* < 0.05 as the significance threshold.

## Results

### Component Analysis of AIMP

**Figure 2:**
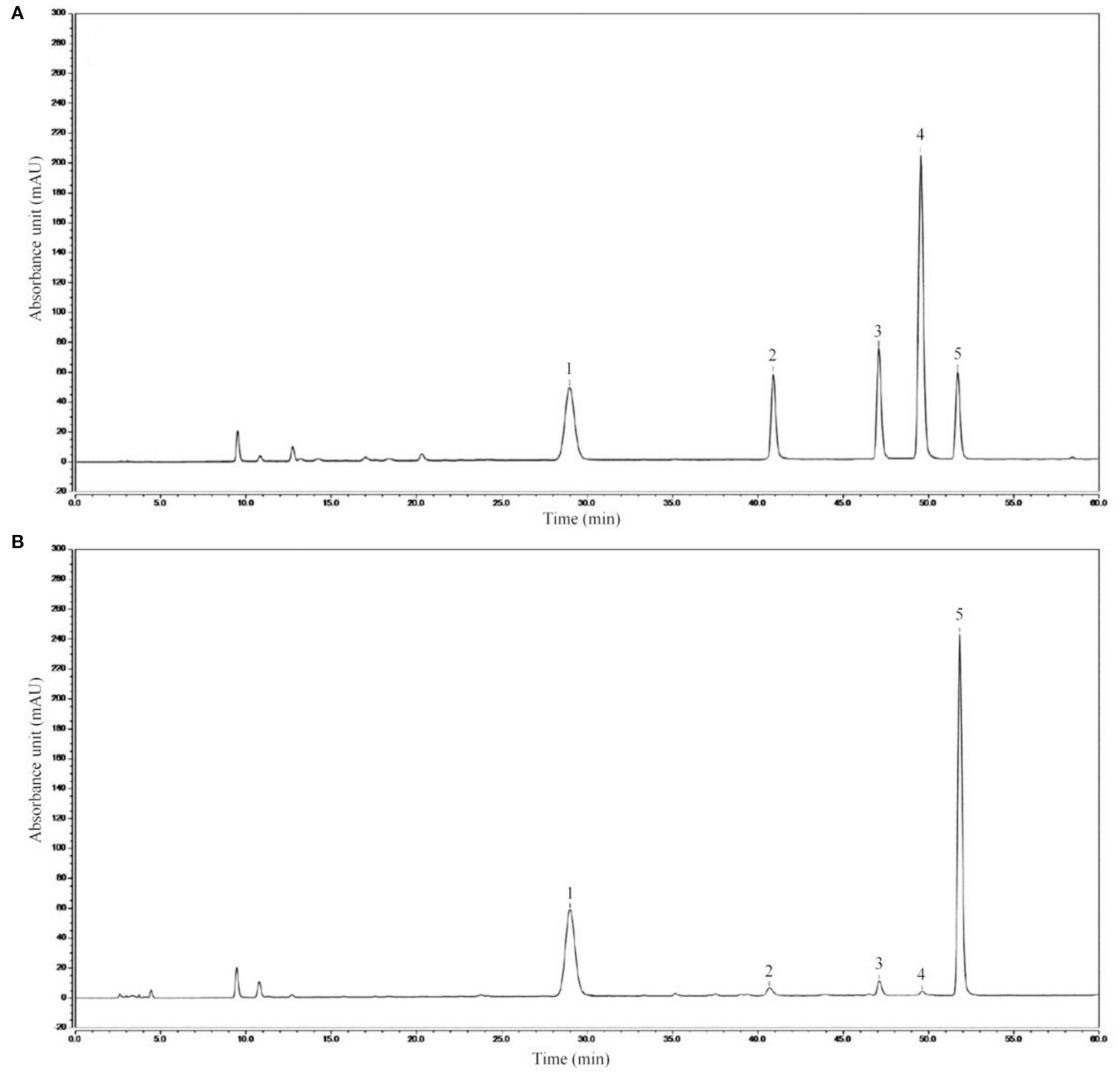
AIMP composition. **(A)** Standard chromatograms; **(B)** AIMP chromatograms. 1: mannitol, 2: ribose, 3: anhydrous glucose, 4: xylose, 5: fucose.

The high-performance liquid chromatography analysis results revealed AIMP to be primarily composed of mannitol (236.12 ± 1.05 mg), ribose (15.02 ± 0.56 mg), anhydrous glucose (14.19 ± 0.31 mg), xylose (17.82 ± 0.36 mg), and fucose (462.33 ± 1.11 mg, Figure 2), with the fucose content being the highest.

### Toxicity, Weight and Organ Index

The model group mice exhibited lower thymic, hepatic, splenic, renal, and brain index values than did the mice in the other groups, while these values were maximal in the control group mice (Table 2). AIMP and Vit C treatments were associated with significant improvements in these organ index values in the D-galactose-treated mice (*p* < 0.05), with AIMP-induced improvements being significantly better than Vit C-induced improvements. Together, these findings suggest that AIMP treatment is sufficient to inhibit oxidative damage-induced declines in organ index values in D-galactose-treated mice.

**Table 2:** Organ index values in treated mice (*N* = 10).

**Group**		**Control**	**Model**	**Vit C**	**AIMP-L**	**AIMP-H**
Body	Weight (g)	36.48 ± 3.27^a^	34.78 ± 3.69^a^	35.48 ± 4.08^a^	35.22 ± 3.68^a^	36.29 ± 3.83^a^
Thymus	Weight (mg)	74.05 ± 4.32^a^	31.65 ± 2.17^e^	53.93 ± 3.44^c^	42.81 ± 2.98^d^	62.42 ± 3.79^b^
	Index	2.03 ± 0.20^a^	0.91 ± 0.18^e^	1.52 ± 0.11^c^	1.22 ± 0.08^d^	1.72 ± 0.12^b^
Brain	Weight (mg)	767.17 ± 12.35^a^	374.93 ± 8.20^e^	583.29 ± 6.60^c^	418.77 ± 8.23^d^	657.21 ± 10.36^b^
	Index	21.03 ± 0.42^a^	10.78 ± 0.23^e^	16.44 ± 0.34^c^	11.89 ± 0.32^d^	18.11 ± 0.20^b^
Cardiac	Weight (mg)	191.52 ± 6.79^a^	140.86 ± 4.97^e^	162.14 ± 4.06^c^	148.63 ± 6.22^d^	178.91 ± 6.11^b^
	Index	5.25 ± 0.05^a^	4.05 ± 0.05^e^	4.57 ± 0.06^c^	4.22 ± 0.03^d^	4.93 ± 0.05^b^
Liver	Weight (mg)	1508.45 ± 35.67^a^	942.89 ± 12.36^e^	1172.61 ± 25.32^c^	1100.63 ± 30.25^d^	1360.88 ± 32.09^b^
	Index	41.35 ± 0.31^a^	27.11 ± 0.22^e^	33.05 ± 0.26^c^	31.25 ± 0.24^d^	37.50 ± 0.29^b^
Spleen	Weight (mg)	161.61 ± 6.12^a^	70.60 ± 5.31^e^	123.12 ± 7.10^c^	99.67 ± 7.85^d^	142.62 ± 4.33^b^
	Index	4.43 ± 0.24^a^	2.03 ± 0.20^e^	3.47 ± 0.25^c^	2.83 ± 0.19^d^	3.93 ± 0.21^b^
Kidney	Weight (mg)	542.82 ± 5.98^a^	279.98 ± 9.33^e^	399.50 ± 6.92^c^	348.33 ± 6.32^d^	472.13 ± 5.12^b^
	Index	14.88 ± 0.11^a^	8.05 ± 0.09^e^	11.26 ± 0.16^c^	9.89 ± 0.16^d^	13.01 ± 0.14^b^

*Data are means ± standard deviations (N = 10/group)*.

a−e *Different superscript letters within a row correspond to significant differences (p < 0.05; Duncan's multiple range test). Vc: mice treated with 100 mg/kg vitamin C; AIMP-L: mice treated with 50 mg/kg AIMP; AIMP-H: treatment with 100 mg/kg AIMP*.

### Histology Analysis

**Figure 3:**
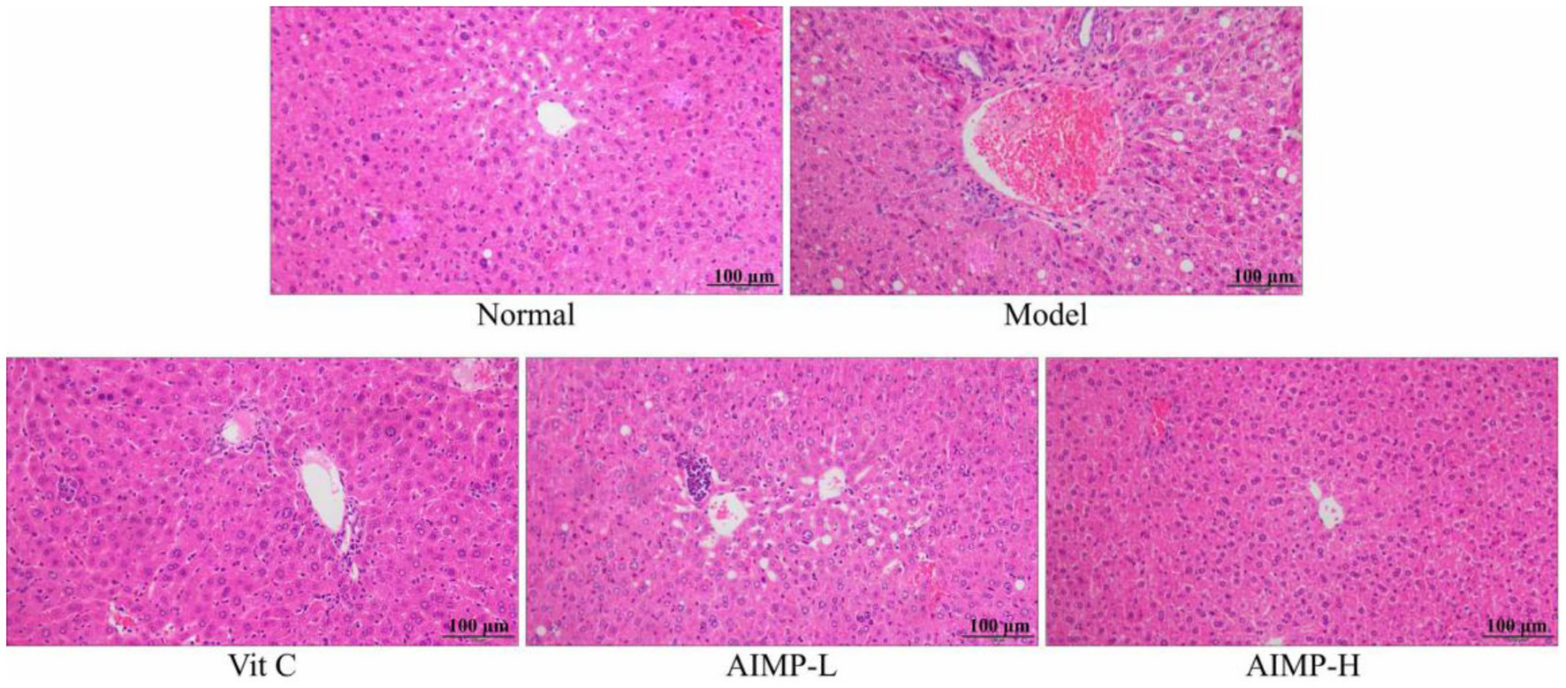
Pathological assessment of H&E-stained hepatic sections from treated mice. Magnification 100×.

The control group mice exhibited standard hepatic histology, with hepatocytes being radially arranged in an ordered fashion around the central vein and with no evidence of inflammatory cell infiltration (Figure 3). In contrast, the model group mice exhibited erratic and disordered hepatocyte arrangement, with irregular cell morphological findings, such as damaged nuclei, swelling, and the breakdown of the barriers between the cells. Substantial inflammatory infiltration was also observed in the liver samples of the D-galactose-treated mice. Treatment with AIMP was associated with the reversal of the D-galactose-induced damage, with less cellular disorder being evident in the hepatic sections from the mice administered this polysaccharide mixture. AIMP-H treatment resulted in maximal preservation of hepatic cell integrity, with the samples from the AIMP-H-treated mice being most similar to those from the control group mice.

**Figure 4:**
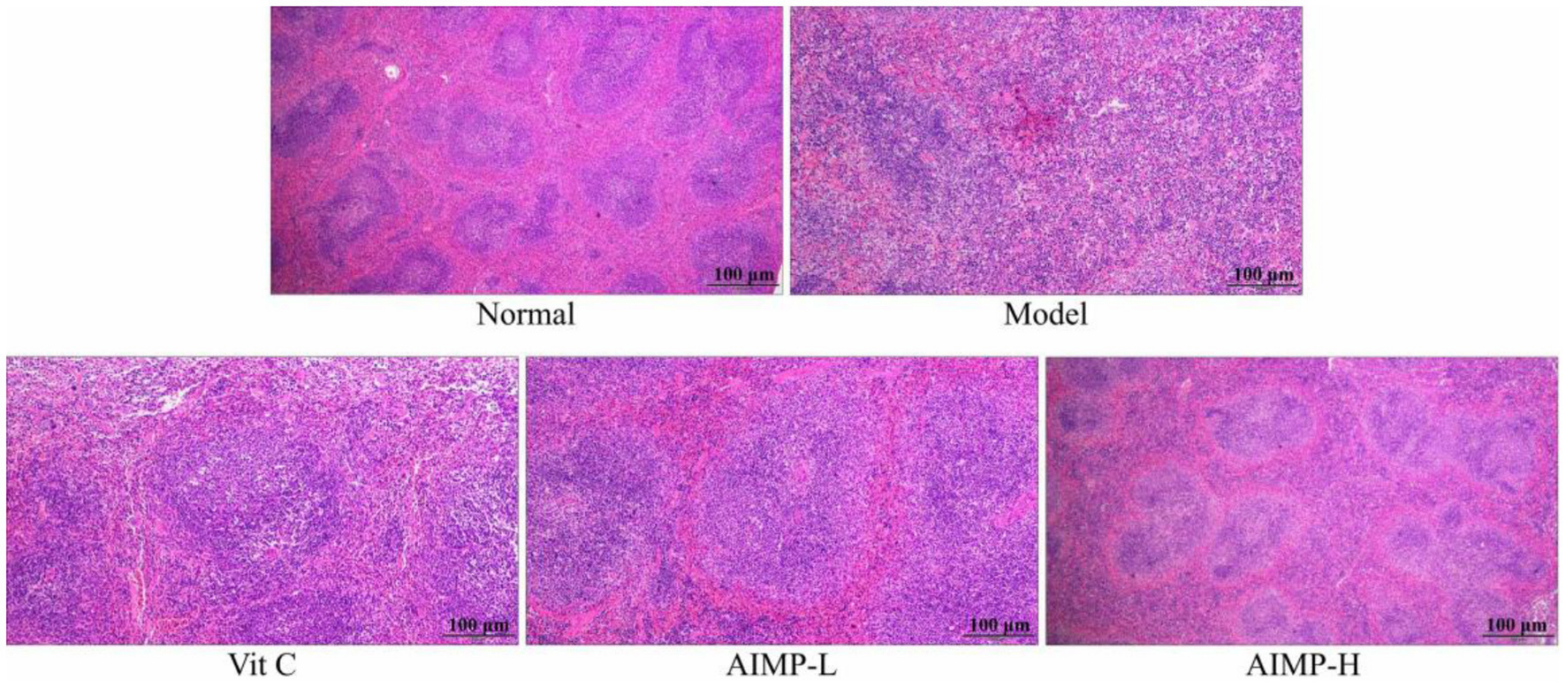
Pathological assessment of H&E-stained splenic sections from treated mice. Magnification 100×.

The control group mice also exhibited normal splenic architecture, as evidenced by clearly ordered cells and regular tissue structure (Figure 4). In contrast, this ordered structure was absent in the D-galactose-treated mice in the model group. The model mice exhibited irregular morphological findings, expansion of the red pulp medullary sinuses, reduced numbers of white pulp lymphocytes, and sparser cell arrangement. AIMP treatment was associated with the reversal of the D-galactose-induced changes in splenic morphology. Together, these results suggest that AIMP treatment is sufficient to inhibit D-galactose-induced oxidative tissue damage in mice, and that this polysaccharide mixture is more beneficial than Vit C, at least in this experimental context.

### Assessment of Oxidative Stress Markers

#### Assessment of ALT, AST, and AKP Levels in the Serum of the Treated Mice

The control group mice showed the lowest ALT, AST, and AKP serum levels, while these indexes were highest in the model group mice (Table 3). The ALT, AST, and AKP levels of the AIMP-H mice were closest to those of the control group mice, and these levels in the AIMP-H mice were significantly lower than those in the Vit C, AIMP-L, and model group mice (*p* < 0.05).

**Table 3:** Serum ALT, AST, and AKP levels in treated mice (*N* = 10).

**Group**	**ALT (U/L)**	**AST (U/L)**	**AKP (U/L)**
Control	15.35 ± 1.20^e^	11.05 ± 1.05^e^	31.22 ± 3.12^e^
Model	61.37 ± 2.12^a^	57.66 ± 1.97^a^	93.59 ± 5.28^a^
Vit C	36.02 ± 1.78^c^	29.60 ± 1.55^c^	62.80 ± 4.41^c^
AIMP-L	45.62 ± 2.01^b^	42.56 ± 2.38^b^	46.08 ± 3.92^b^
AIMP-H	28.33 ± 1.62^d^	20.36 ± 1.73^d^	75.10 ± 3.36^d^

*Data are means ± standard deviations (N = 10/group)*.

a−e *Different superscript letters within a column correspond to significant differences (p < 0.05; Duncan's multiple range test). Vc: mice treated with 100 mg/kg vitamin C; AIMP-L: mice treated with 50 mg/kg AIMP; AIMP-H: treatment with 100 mg/kg AIMP*.

#### Expression of nNOS, eNOS, and iNOS in Hepatic and Splenic Samples

**Figure 5:**
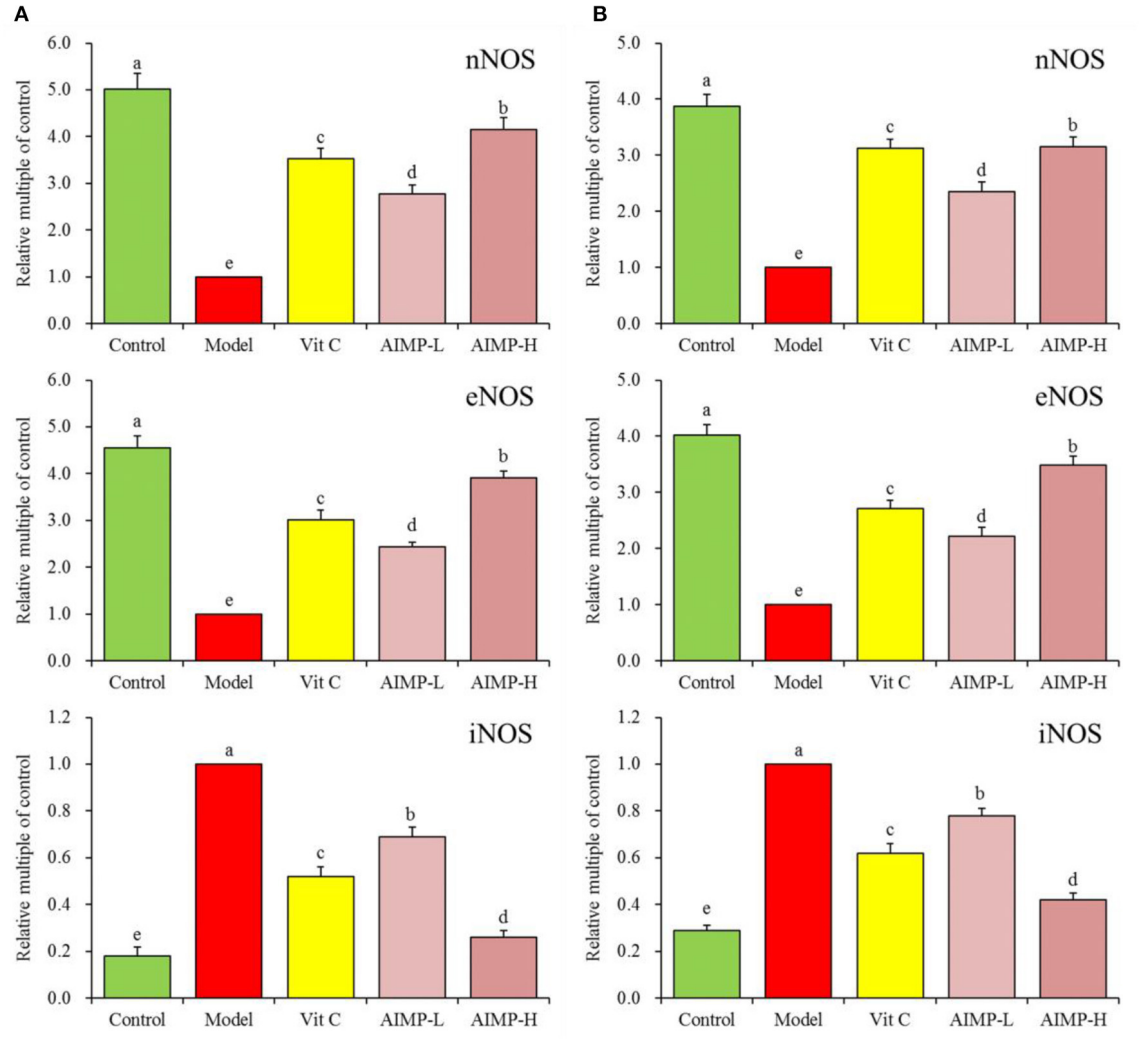
Expression of hepatic **(A)** and splenic **(B)** nNOS, eNOS, and iNOS at the mRNA level in mice treated as indicated. ^a−*e*^Different superscript letters within a column correspond to significant differences (*p* < 0.05; Duncan's multiple range test).

**Figure 6:**
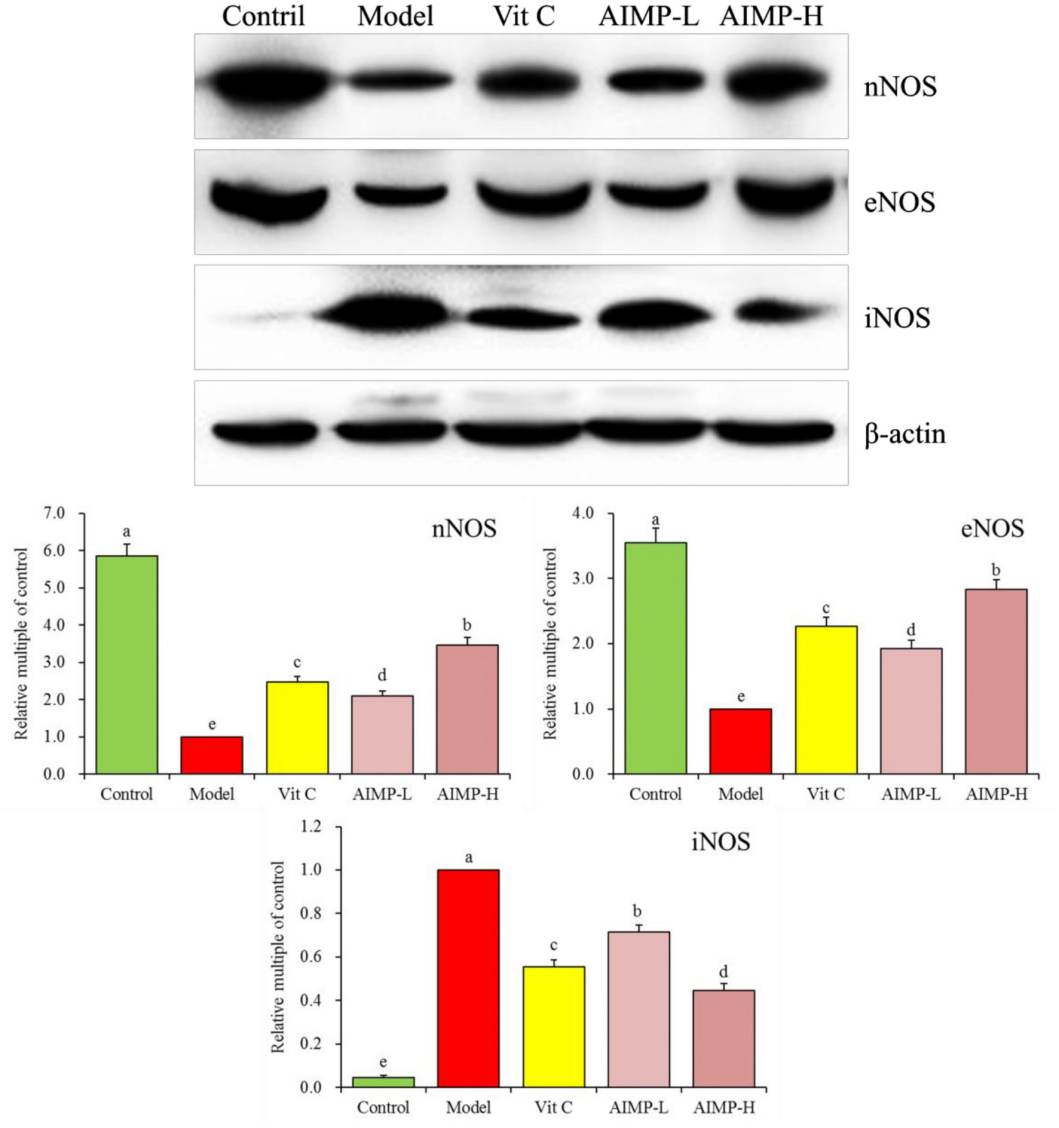
Expression of hepatic nNOS, eNOS, and iNOS at the protein level in mice treated as indicated. ^a−e^Different superscript letters within a column correspond to significant differences (*p* < 0.05; Duncan's multiple range test).

We next evaluated the neuronal nitric oxide synthase (nNOS), endothelial nitric oxide synthase (eNOS), and inducible nitric oxide synthase (iNOS) expression levels in the hepatic and splenic samples of the mice (Figures 5, 6). We found that relative to the mice in the other groups, the mice in the control group exhibited significant increases in the mRNA levels of nNOS and eNOS in both the spleen and liver as well as significant increases in the protein levels of nNOS and eNOS in the liver (*p* < 0.05). The mRNA levels of iNOS in the spleen and liver and the protein levels of iNOS in the liver of the mice in the control group were significantly lower than those in the mice in any other group (*p* < 0.05). D-galactose treatment was associated with significant reductions in nNOS and eNOS expression levels and with significant increases in iNOS expression levels in both hepatic and splenic tissues. AIMP treatment was sufficient to significantly reverse the D-galactose-induced changes in all nitric oxide synthase (NOS) expression levels (*p* < 0.05), and it was more efficacious than an equivalent dose of Vit C treatment.

#### Expression of Cu/Zn-SOD, Mn-SOD, and CAT in Hepatic and Splenic Samples

**Figure 7:**
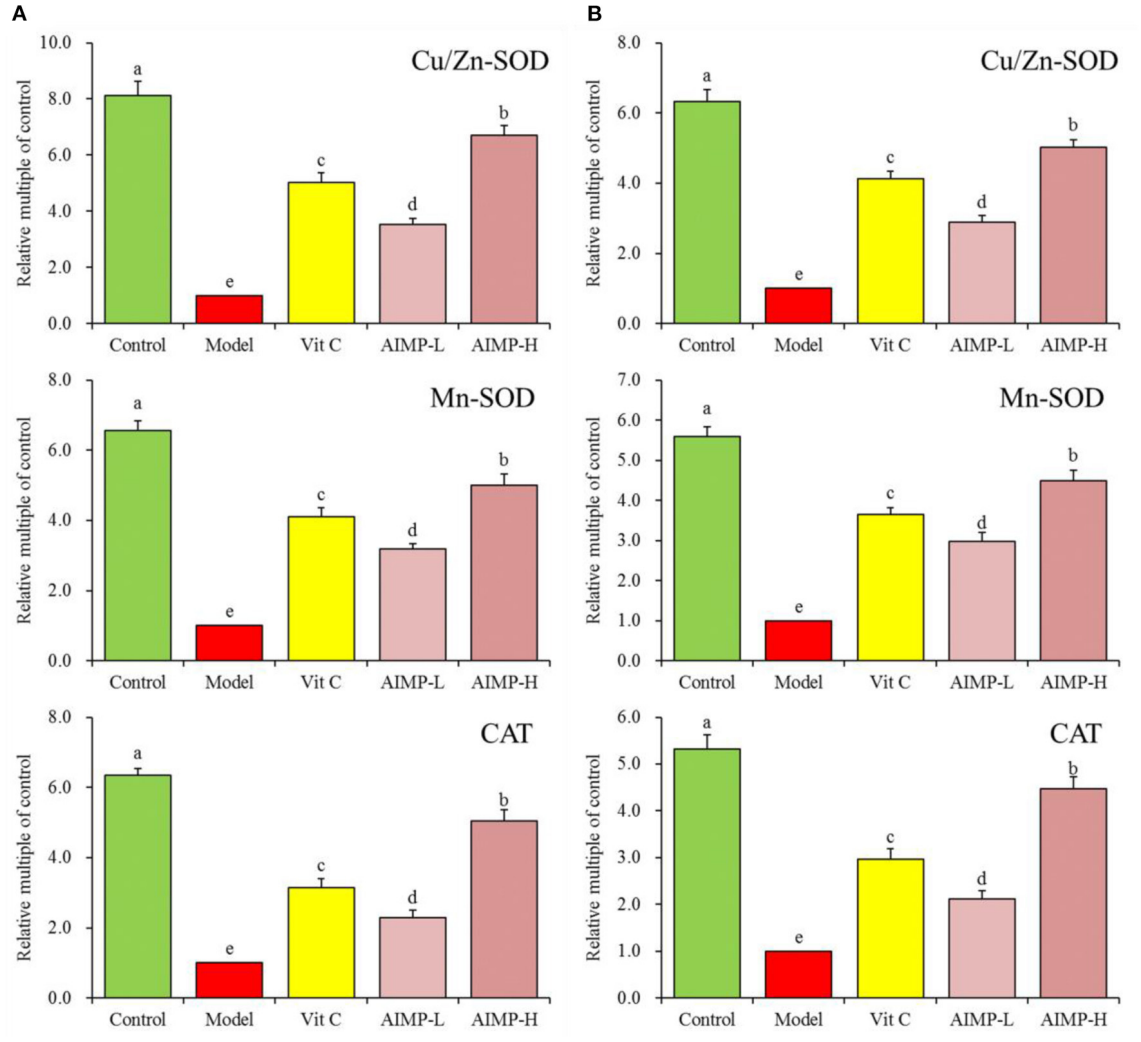
Expression of hepatic **(A)** and splenic **(B)** Cu/Zn-SOD, Mn-SOD, and CAT at the mRNA level in mice treated as indicated. ^a−e^Different superscript letters within a column correspond to significant differences (*p* < 0.05; Duncan's multiple range test).

**Figure 8:**
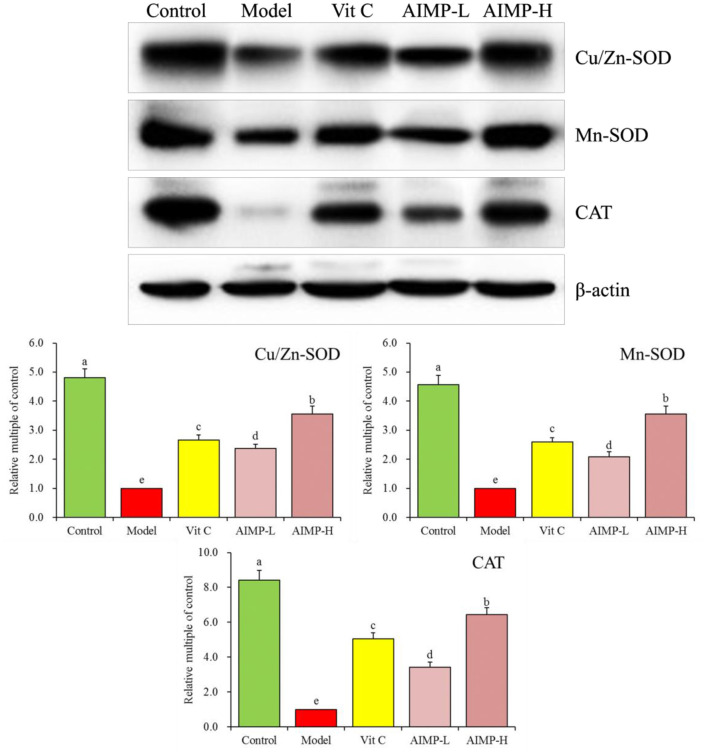
Expression of hepatic Cu/Zn-SOD, Mn-SOD, and CAT at the protein level in mice treated as indicated. ^a−e^Different superscript letters within a column correspond to significant differences (*p* < 0.05; Duncan's multiple range test).

We also evaluated the cuprozinc-superoxide dismutase (Cu/Zn-SOD), manganese superoxide dismutase (Mn-SOD), and catalase (CAT) mRNA levels in the hepatic and splenic samples and protein levels in the hepatic samples of the mice (Figures 7, 8). We found maximal expression levels of these markers in the control group mice. In contrast, the expression levels of these markers were downregulated in the model group mice. AIMP and Vit C treatments were associated with significant increases in hepatic and splenic CAT, Cu/Zn-SOD, and Mn-SOD expression levels in the D-galactose-treated mice (*p* < 0.05), with the levels in the AIMP-H-treated mice being closest to those in the control group mice.

#### Assessment of Hepatic and Splenic HO-1, Nrf2, γ-GCS, and NQO1 in the Treated Mice

**Figure 9:**
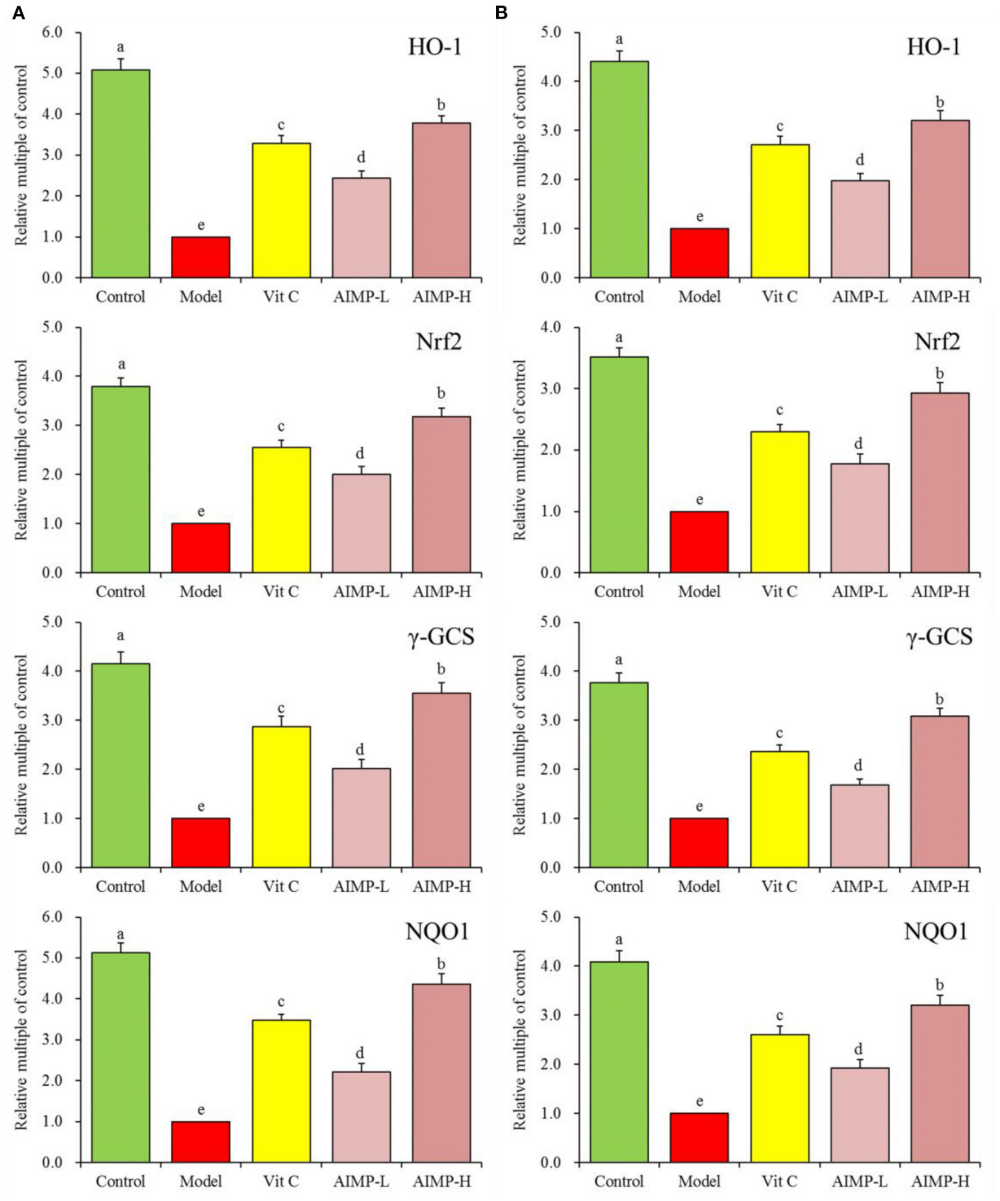
Expression of hepatic **(A)** and splenic **(B)** HO-1, Nrf2, γ-GCS, and NQO1 at the mRNA level in mice treated as indicated. ^a−e^Different superscript letters within a column correspond to significant differences (*p* < 0.05; Duncan's multiple range test).

**Figure 10:**
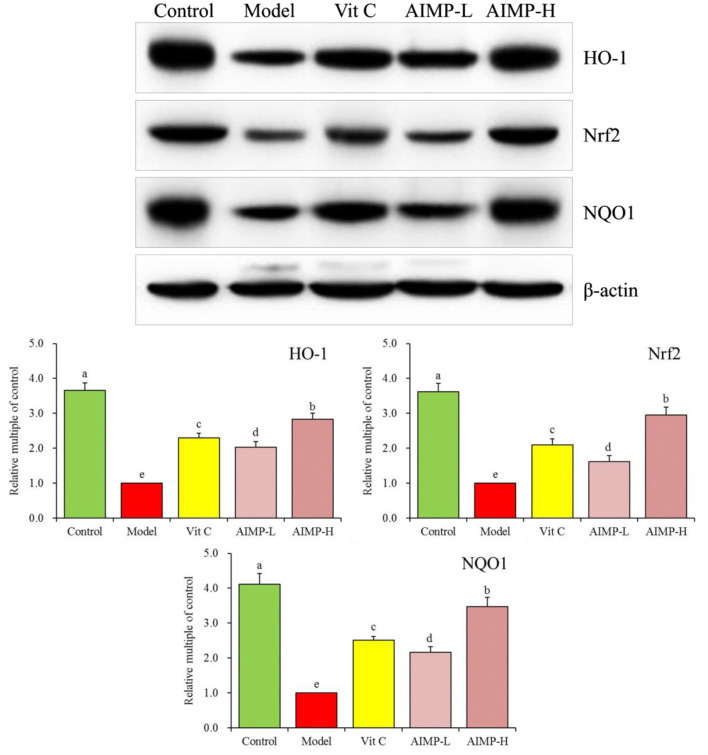
Expression of hepatic HO-1, Nrf2, and NQO1 at the protein level in mice treated as indicated. ^a−e^Different superscript letters within a column correspond to significant differences (*p* < 0.05; Duncan's multiple range test).

We also evaluated the HO-1, nuclear factor erythroid 2-related factor 2 (Nrf2), γ-glutamylcysteine synthetase (γ-GCS), and NAD(P)H dehydrogenase [quinone] 1 (NQO1) mRNA levels in the hepatic and splenic samples and protein levels in the hepatic samples of the mice (Figures 9, 10). We found that relative to the mice in the other groups, these genes were expressed at significantly elevated levels in the mice in the control group (*p* < 0.05). In contrast, the model group mice exhibited the lowest levels of splenic HO-1, Nrf2, γ-GCS, and NQO1 expression. The levels of these genes in both the hepatic and splenic samples rose significantly in response to AIMP-H treatment, with the beneficial effects of this polysaccharide solution being superior to those of the AIMP-L and Vit C preparations (*p* < 0.05).

### Assessment of Antioxidant Enzymes

Relative to the mice in the other treatment groups, the mice in the model group exhibited significant reductions in SOD and GSH-Px activity in the spleen, liver, and serum (Table 4), while the NO and MDA levels in the mice in the model group were higher than those in the mice in the other groups. The mice in the control group had maximal SOD, GSH-Px, and glutathione activity and the lowest NO and MDA levels. AIMP treatment was associated with significant increases in SOD, GSH-PX, and glutathione activity and significant reductions in MDA and NO levels (*p* < 0.05). Indeed, AIMP treatment resulted in these values being relatively close to those in the control group mice, and AIMP treatment was significantly better than Vit C treatment in terms of the ability to remediate the D-galactose-induced changes in antioxidant activity (*p* < 0.05).

**Table 4:** The NO, SOD, GSH-Px, GSH, and MDA levels of serum, hepatic tissue and splenic tissue in treated mice (*N* = 10).

**Group**	**NO (μmol/L)**	**SOD (U/mL)**	**GSH-Px (U/mL)**	**GSH (mg/L)**	**MDA (nmol/mL)**
Normal	12.51 ± 0.25^e^	244.89 ± 11.29^a^	225.62 ± 12.08^a^	51.08 ± 3.68^a^	3.12 ± 0.32^e^
Model	56.51 ± 1.23^a^	56.36 ± 5.98^e^	70.19 ± 7.73^e^	8.02 ± 0.79^e^	35.67 ± 1.12^a^
Vit C	32.45 ± 1.71^c^	165.30 ± 12.65^c^	139.87 ± 10.61^c^	31.88 ± 2.03^c^	9.97 ± 0.54^c^
AIMP-L	40.89 ± 1.69^b^	88.71 ± 10.25^d^	92.79 ± 9.11^d^	21.05 ± 1.88^d^	18.96 ± 1.08^b^
AIMP-H	18.46 ± 1.08^d^	210.38 ± 11.20^b^	188.10 ± 11.50^b^	41.10 ± 2.29^b^	5.67 ± 0.42^d^
**Group**	**NO (μmol/g protein)**	**SOD (U/mgprot)**	**GSH-Px (U/mgprot)**	**GSH (mg/ gprot)**	**MDA (nmol/mgprot)**
Normal	2.16 ± 0.16^e^	94.65 ± 5.26^a^	181.09 ± 9.75^a^	14.55 ± 0.74^a^	1.18 ± 0.15^e^
Model	11.05 ± 0.75^a^	18.79 ± 2.61^e^	46.85 ± 4.91^e^	1.68 ± 0.36^e^	10.36 ± 0.63^a^
Vit C	4.98 ± 0.42^c^	68.47 ± 3.82^c^	118.63 ± 8.13^c^	6.79 ± 0.49^c^	4.33 ± 0.23^c^
AIMP-L	8.32 ± 0.35^b^	42.58 ± 3.08^d^	88.75 ± 4.23^d^	3.66 ± 0.27^d^	6.91 ± 0.28^b^
AIMP-H	3.44 ± 0.22^d^	80.69 ± 2.91^b^	159.81 ± 6.79^b^	10.25 ± 0.62^b^	2.01 ± 0.20^d^
Normal	1.69 ± 0.16^e^	89.12 ± 8.69^a^	135.10 ± 11.68^a^	9.62 ± 0.70^a^	0.60 ± 0.11^e^
Model	9.88 ± 0.60^a^	15.97 ± 4.11^e^	31.20 ± 3.66^e^	1.56 ± 0.47^e^	7.32 ± 0.45^a^
Vit C	4.23 ± 0.36^c^	56.51 ± 6.03^c^	86.36 ± 7.25^c^	5.33 ± 0.63^c^	2.97 ± 0.22^c^
AIMP-L	6.99 ± 0.41^b^	29.65 ± 4.28^d^	51.58 ± 5.44^d^	3.82 ± 0.32^d^	4.89 ± 0.36^b^
AIMP-H	2.06 ± 0.22^d^	70.36 ± 5.12^b^	114.59 ± 9.71^b^	7.03 ± 0.38^b^	1.12 ± 0.16^d^

*Data are means ± standard deviations (N = 10/group)*.

a−e *Different superscript letters within a column correspond to significant differences (p < 0.05; Duncan's multiple range test). Vc: mice treated with 100 mg/kg vitamin C; AIMP-L: mice treated with 50 mg/kg AIMP; AIMP-H: treatment with 100 mg/kg AIMP*.

## Discussion

Antarctic ice algae (*Chlamydomonas* sp.) have the characteristics of common algae, and they are rich in polysaccharides (20). Seaweed cells contain certain polysaccharides that play important roles in controlling cell division, regulating cell growth, and maintaining the normal metabolism of living organisms (21). A variety of seaweed polysaccharides have biological activities, including scavenging free radicals *in vitro*, protecting cells from oxidative damage, and inhibiting oxidative damage in animals (22–24). In this study, mannitol, ribose, anhydrous glucose, xylose, and fucose were found in AIMP for the first time. We also observed AIMP's effects on chemically induced oxidative damage in mice, and we analyzed the relationships between its components and its effects. Mannitol is readily absorbed by the human gastrointestinal tract. After intake, however, it is partially metabolized and excreted in the urine. It can be used to treat nephropathy and to alleviate intracranial and intraocular pressure (25). Ribose is an RNA component that is essential for normal physiology, and as a reducing sugar, it also exhibits antioxidant properties (26). Glucose is an essential energy source for cells and a metabolic intermediate (27). Xylose is a monosaccharide with anti-fungal and anti-bacterial properties that aids in intestinal probiotic growth (28). Fucose is another physiologically active monosaccharide that is associated with inhibiting cancer metastasis, promoting immune functionality, and treating infections of the respiratory tract (29). The high fucose levels in AIMP are likely associated with the antioxidant effects of AIMP preparations, with the other four monosaccharides also contributing to this efficacy.

Organ weights and organ index values are commonly used to evaluate physiological changes *in vivo* in animal model systems, and they are thus optimal readouts for oxidative damage (30). In particular, the effect of oxidative stress on the liver and spleen is particularly obvious, and also in the thymus and brain. The index of these organs can effectively test the degree of oxidative damage (31–33). In the present study, we found that D-galactose treatment in mice was sufficient to induce significant decreases in organ index values that were consistent with oxidative damage. Importantly, AIMP treatment was sufficient to prevent this oxidative damage, with AIMP-treated mice exhibiting organ index values similar to those of healthy mice.

Oxidative stress induced by ROS is the common pathophysiological basis of many liver diseases (34). ALT, AST, and AKP are important indicators of liver function. The liver is the main organ that produces AKP. When the liver is damaged, hepatocytes overproduce AKP and flow back into the blood through lymphatic channels and hepatic sinuses (35). ALT and AST are mainly distributed in hepatocytes. When hepatocytes are part of necrotic tissue, ALT and AST are released into the blood circulation, resulting in elevated serum enzyme levels, which are positively correlated with the degree of liver tissue damage (19). We found that AIMP reduced serum AST, ALT, and AKP levels and thereby alleviated liver injury caused by oxidative stress.

The activation of NOS and the production of NO will trigger the production of O_2_ and OH, leading to cell damage. The interaction of no and ${\text{O}}_{2}^{-}$ promotes the formation of OH^−^ and ${\text{NO}}_{2}^{-}$ (36). Overexpression of iNOS and NO production will damage stress resistance, thus promoting brain aging and aggravation of inflammation (37, 38). eNOS can inhibit oxidative stress-induced vascular aging and protect tissues (39). In addition, nNOS can protect myocardium, smooth muscle and skeletal muscle from oxidative damage (40, 41). iNOS is a proinflammatory cytokine and exogenous antioxidant inhibitor (42, 43). Oxidative stress in the organism production of a large amount of ROS can promote the activation of iNOS, which can affect the expression levels of nNOS and eNOS in tissues, seaweed polysaccharides can also play a role in inhibiting oxidative stress by regulating these expression (44–46). Herein, we found that AIMP treatment was sufficient to modulate the levels of NO, nNOS, eNOS, and iNOS in D-galactose-treated mice, helping return these levels to those found in the normal healthy (control group) mice by mitigating D-galactose-induced oxidative damage.

SOD can prevent the accumulation of superoxide radicals, thus preventing oxidative damage (47). Oxidative damage, in turn, resulted in reduced levels of two major types of SOD (Cu/Zn SOD and Mn SOD) in the affected tissues (48, 49). CAT mainly exists in cytoplasm and mitochondria and is an antioxidant enzyme (50). Under normal conditions, excessive ROS produced by cells is rapidly neutralized by the activities of CAT, SOD, GSH-Px and other enzymes (51). Glutathione is capable of serving as a scavenger for free radicals within cells, thus reducing ROS-associated lipid peroxidation (52, 53). MDA is a lipid peroxide produced in response to oxidative damage, with MDA levels thus offering insight into *in vivo* oxidation levels (54). Herein, we found that AIMP treatment restored SOD, CAT, glutathione, MDA, and GSH-Px levels in the mice administered D-galactose to levels closer to those in the healthy mice. This result suggests that AIMP preparations can mitigate oxidative damage *in vivo*.

HO-1 is a stress protein that is important for heme metabolism, and it is associated with key anti-inflammatory and antioxidant activities. HO-1 exerts cardioprotective and neuroprotective effects owing to its antioxidant activity in the context of atherosclerosis, hypertension, Alzheimer's disease, and other neuronal diseases (55). Nrf2 is a key modulator of vascular endothelial integrity, and its altered functionality in response to oxidative stress can promote HO-1 expression as well as CAT and SOD transcription, thus improving antioxidant activity (56). Nrf2 also regulates γ-GCS, and large quantities of excess ROS lead to Nrf2 activation and high levels of γ-GCS expression, which in turn stimulate glutathione synthesis (57). Under homeostatic conditions, Nrf2 is bound by Keap1, but in response to oxidative stress, these two proteins dissociate and Nrf2 enters the nucleus, where it can bind to ARE elements to drive the expression of the antioxidant enzyme NQO1. This Nrf2/NQO1 signaling pathway is a key means whereby certain bioactive compounds exert their antioxidant effects (58). Herein, we found that AIMP treatment was associated with Nrf2 activity and enhanced HO-1, γ-GCS, and NQO1 levels, thereby protecting tissues from oxidative damage.

Antarctic ice algae are from the high latitudes and cold areas of Antarctica, and the algae that survive this harsh environment (low temperature, low light, and high ultraviolet radiation) contain more antioxidants than algae from areas with temperate temperatures. The protein, polysaccharide, and polyunsaturated fatty acid levels in Antarctic ice algae are higher than those in algae from areas with temperate temperatures (59). Antarctic ice algae are rich in antioxidants, and the antioxidant effect may be better than that of algae from areas with temperate temperatures. The polysaccharides of Antarctic ice microalgae were analyzed in this study. The high-quality monosaccharide composition was an important reason for the algae's good antioxidant properties, which was also proved by the results of the animal experiments. In addition, Antarctic ice algae taste good in traditional foods. However, its components may be affected in transportation and processing, which may affect its functional function. Therefore, a more simple and effective extraction method needs to be studied. At the same time, the AIMP is mainly composed of five kinds of monosaccharides, but the more complex molecular structure and proportion need further component analysis.

Marine microalgae diatoms, chlorophytes, rhodophytes and dinoflagellates all contain polysaccharides, which are composed of galactose, mannose, xylose and rhamnose. Other marine microalgae, such as *P. cruentum, P. purpureum, Rhodella reticulata* and *R. maculate*, contain 3-O-methyl-xylose, 3-O-methyl-rhamnose and 4-O-methyl-galactose in addition to the above monosaccharides (58, 59). Marine microalgae polysaccharides can protect against oxidative stress by regulating Nrf2/HO-1 and apoptosis-related signaling pathways, so as to repair oxidative stress-induced damage (60). This study showed that AIMP also had a certain role in the activation of the Nrf2/HO-1 pathway, suggesting that it may play a regulatory role and also have an antioxidant effect. At the same time, AIMP showed some similarities with other seaweed polysaccharides. The mechanism of AIMP was similar to that of other seaweed polysaccharides, but the differences between the mechanisms need further study.

## Conclusions

The results of this study showed that AIMP prevented oxidative damage induced by D-galactose in mice. The serum, hepatic, and splenic findings in the AIMP-treated mice were closer to those in the normal mice (control group) than to those in the Vit C-treated mice. Our results highlight the importance of future studies of the *in vivo* bioactivity of AIMP preparations and derivatives thereof. We conducted a preliminary evaluation of the monosaccharides comprising AIMP, but future work is needed to clarify how these individual components contribute to the overall physicochemical properties of AIMP preparations. The activity of AIMP in human body must also be evaluated, because the utilization of this polysaccharide in human digestive system may be different from that in mice. This study was a preliminary investigation of the components and antioxidant capacity of AIMP. The mechanism of action of AIMP and its impact on the human body have not yet been fully elucidated. Additional preclinical and clinical studies of AIMP will be invaluable for our understanding of the potential pharmacological utility of this natural marine resource. In depth research is beneficial to the development of effective nutrients or drugs.

## Data Availability Statement

The original contributions presented in the study are included in the article/supplementary material, further inquiries can be directed to the corresponding author/s.

## Ethics Statement

The protocol for these experiments was approved by the Ethics Committee of Chongqing Collaborative Innovation Center for Functional Food (201905030B), Chongqing, China. The experimental process was in accordance with 2010/63/EU directive.

## Author Contributions

RY and LD performed the majority of the experiments and wrote the manuscript. JM and CL contributed to the data analysis. XZ and FT designed and supervised the study, and checked the final manuscript. All authors contributed to the article and approved the submitted version.

## Conflict of Interest

The authors declare that the research was conducted in the absence of any commercial or financial relationships that could be construed as a potential conflict of interest.
